# Editorial

**Published:** 1840-01-18

**Authors:** 


THE MEDICAL EXAMINER.

PHILADELPHIA, JANUARY 18, 1840.

### DR. MAY'S INTRODUCTORY LECTURE.

We have received the lecture by Dr. May, introductory to a course of lectures on Anatomy in the Washington Medical School. We are much pleased with it, not only for its merits, as a well written lecture, but because it lays a just stress upon pathological anatomy as an aid in the cultivation of medical science.

One word in reference to introductory lectures—We are greatly obliged to our friends for copies of their introductories, which we shall not fail to notice, although, perhaps, briefly. We are sure that they will take this mode of announcement in good part; it is the best and most genuine compliment which we can pay them. An introductory does not, as a general rule, admit of analysis, and we believe that the lecturers will agree with us that it is not a suitable occasion for a personal eulogium. Of course, if an *Introductory* should differ from the usual lectures classed under this title, we would analyse it like any other memoir of a similar kind. But then its character is totally changed, perhaps not to the benefit of the hearers, who are generally best satisfied with the lectures which may be personally most interesting to themselves, but least so to the profession.

### YELLOW FEVER OF THE WEST INDIES.

We commence the publication of the Memoir of Dr. Rufz, a resident of the island of Martinique. It was sent to us by the author a short time since, with permission to publish it entire, or to extract from it such portions as we might prefer. The Memoir is of so finished a character, that we believed it would be doing injustice to the members of the profession, to cut it in pieces, for the purpose of sparing some details not immediately connected with the subject. The treatment of the disease by large and frequently repeated depletion, is not, we believe, the method that was regarded most effectual in the southern states; but we imagine that yellow fever, like all diseases having a natural termination, will be found to require different methods of treatment, in different individuals and epidemics. At least the evidence of Dr. Rufz is decidedly in favor of the mode which he recommends. The treatment is necessarily reserved for a subsequent number.

### DR. COATES' LECTURES.

We have attended many of Dr. Coates' popular lectures on physiology, and can cheerfully concur with the resolutions which we copy from the National Gazette of this city. Dr. Coates may visit other parts of the country, and we are sure that he will be every where heard with as much pleasure and profit as at Philadelphia.

"At the close of Dr. Reynell Coates' lectures on Saturday evening, January 11th, the audience was requested to remain; and, after the lecturer had withdrawn, the meeting was organized by appointing Professor C. D. Cleveland, chairman, and Dr. Henry Bond, secretary. Dr. Caspar Wistar then offered the following preamble and resolutions, which were unanimously adopted.

Whereas, Dr. Reynell Coates having now concluded his course of Lectures on Physiology, this class are unwilling to separate without offering some testimonial of their respect for the lecturer, and expressing their satisfaction with this first attempt to render an important branch of natural science at once instructive and entertaining to a popular audience: therefore,

*Resolved*, That we have listened with great satisfaction to the highly interesting lectures just delivered by Dr. Coates, and that the lecturer has not only fulfilled the promise of his prospectus, and the anticipation of his friends, but has evinced signal ability in elucidating the difficult doctrines of Physiology.

*Resolved*, That an intimate acquaintance with the science, an uncommon command of the most appropriate and perspicuous language, delivered in the best manner for a teacher of science, and a happy facility of illustrating a subject by facts and specimens, entitle Dr. Coates, in the estimation of this class, to rank among the most instructive and entertaining lecturers of Philadelphia.

*Resolved*, That we take pleasure in thus bearing testimony to the talents and acquirements which the lecturer has evinced throughout his course, and in publicly expressing our acknowledgments to him for his eminently successful effort for our instruction and gratification.

*Resolved*, That a copy of these resolutions, signed by the Chairman and Secretary, be presented to Dr. Coates, and that they be published in the newspapers of the city.

C. D. Cleveland, Chairman.
Henry Bond, Secretary.

In our next will appear a notice of Dr. Gross' Pathological Anatomy.

The organization of the Philadelphia College of Medicine, which was incorporated at the last session of the Pennsylvania Legislature, is nearly complete. No faculty can be attached to this body; its object will now be to appoint a Board of Examiners, and institute such regulations as will give efficiency to the college.

